# Construction of the Digital Health Equity-Focused Implementation Research Conceptual Model - Bridging the Divide Between Equity-focused Digital Health and Implementation Research

**DOI:** 10.1371/journal.pdig.0000509

**Published:** 2024-05-22

**Authors:** Lisa L. Groom, Antoinette M. Schoenthaler, Devin M. Mann, Abraham A. Brody

**Affiliations:** 1 Rory Meyers College of Nursing, New York University, New York, New York, United States of America; 2 Medical Center Information Technology Department of Health Informatics, New York University Langone Health, New York, New York, United States of America; 3 Institute for Excellence in Health Equity, New York University Langone Health, New York, New York, United States of America; 4 Department of Population Health, New York University Grossman School of Medicine, New York, New York, United States of America; 5 Division of Geriatric Medicine and Palliative Care, New York University Grossman School of Medicine, New York, New York, United States of America; Washington University in Saint Louis, UNITED STATES

## Abstract

Digital health implementations and investments continue to expand. As the reliance on digital health increases, it is imperative to implement technologies with inclusive and accessible approaches. A conceptual model can be used to guide equity-focused digital health implementations to improve suitability and uptake in diverse populations. The objective of this study is expand an implementation model with recommendations on the equitable implementation of new digital health technologies. The Digital Health Equity-Focused Implementation Research (DH-EquIR) conceptual model was developed based on a rigorous review of digital health implementation and health equity literature. The Equity-Focused Implementation Research for Health Programs (EquIR) model was used as a starting point and merged with digital equity and digital health implementation models. Existing theoretical frameworks and models were appraised as well as individual equity-sensitive implementation studies. Patient and program-related concepts related to digital equity, digital health implementation, and assessment of social/digital determinants of health were included. Sixty-two articles were analyzed to inform the adaption of the EquIR model for digital health. These articles included digital health equity models and frameworks, digital health implementation models and frameworks, research articles, guidelines, and concept analyses. Concepts were organized into EquIR conceptual groupings, including population health status, planning the program, designing the program, implementing the program, and equity-focused implementation outcomes. The adapted DH-EquIR conceptual model diagram was created as well as detailed tables displaying related equity concepts, evidence gaps in source articles, and analysis of existing equity-related models and tools. The DH-EquIR model serves to guide digital health developers and implementation specialists to promote the inclusion of health-equity planning in every phase of implementation. In addition, it can assist researchers and product developers to avoid repeating the mistakes that have led to inequities in the implementation of digital health across populations.

## Author summary

Digital health is becoming increasingly prevalent in our society, and it is essential that these technologies are designed with inclusivity and accessibility in mind. There are currently no comprehensive implementation models in digital health geared towards equity. To address this need, we developed a conceptual model called the Digital Health Equity-Focused Research Implementation framework. We completed a comprehensive review of existing literature on digital health implementation and digital health equity. The model merges the findings from this review into the existing Equity-focused Implementation Research model. The resulting digital health equity-focused implementation framework consists of five phases: assessing population health status, planning the program, designing the program, implementing the program, and equity-based outcomes. By promoting inclusivity and accessibility, the framework has the potential to improve the suitability and uptake of digital health technologies in diverse communities. By following this framework, researchers and developers can ensure that digital health equity planning is integrated into every step of the implementation process.

## Introduction

Digital health interventions can provide benefits including reductions in readmissions and improved physical and emotional quality of life health outcomes [1]. In the United States, record amounts of private investment dollars have flowed into digital health startups; in 2020 digital health investments topped $9.2 billion domestically and $14.2 billion globally [2]. Meanwhile, there have been large investments in health technology through federal and state governments, including $80 million in American Rescue Plan funding to strengthen U.S. public health information technology [3]. In addition, the Centers for Medicare and Medicaid Services expanded reimbursement beyond remote patient monitoring, including monitoring non-physiological outcomes such as adherence to physical therapy [4]. In light of this insurance coverage and the evidence that digital health can improve clinical outcomes, reduces costs, and improves patient engagement, it is imperative that research and operational implementors ensure that the interventions are accessible and increase equity rather than widening inequities in outcomes [5].

“Disparity” and “inequity” are distinct concepts–disparity describes a difference, while inequity describes unfairness and injustice [6]. In the U.S. the use of the term disparity ranges from suggesting a value-free difference to a value-laden unfair judgment. This paper will focus on inequities, as inequities are differences that are unjust and should be rectified.

Striking health inequities continue to plague the United States healthcare system. Inequities persist across socioeconomic (SES) levels [7]. Previous work has shown how implicit bias and systemic racism has impacted clinical outcomes for minoritized patients [8]. Implicit bias refers to the unconscious attitudes or stereotypes that affect an individual’s understanding, actions, and decisions in an unintentional manner. This impacts patients through the unconscious biases of healthcare providers and the influence of those biases on assessment and treatment decisions [8]. Systemic racism is associated with poorer physical health, and race is a social determinant of health (SDOH) [9]. SDOH encompass domains that influence health inequities including employment, housing instability, language and literacy, and environmental conditions [10]. Limited access to healthcare services and reduced access to healthier food and lifestyle choices also result in populations experiencing poorer health [10].

Digital health implementations can increase health inequities. Termed the “digital divide,” lack of access to technology have been reported in racial and ethnic minoritized populations, individuals with disabilities, limited English proficiency, living in rural areas, and those with low income [11]. Intervention-generated inequity occurs when an intervention disproportionately benefits advantaged people due to inequalities in access, uptake, adherence, and effectiveness [12]. For example, patients may lack internet access, preventing them from using a patient portal. Furthermore, some patients may have limited internet access through cellular data plans. In remote and rural areas, the available internet access options may be of limited bandwidth. Limited access to internet resources can result in intervention-generated inequity, where patients in underserved areas with limited connectivity may encounter challenges scheduling virtual appointments. Digital health tools that require virtual appointments are then benefitting only persons with sufficient bandwidth. On the other hand, when digital technologies are carefully planned and implemented using an inclusive approach to development, inequities may be diminished [13].

Persistent inequities exist in the development of digital health and other technologies. Technology development has typically included predominantly white user research participants, a systematic bias that could lead to potential harms [14–16]. The lack of a diverse sample during development phases results in flawed products, or products that are difficult or unappealing for minoritized populations to use. Seniors, people with disabilities, and people with limited English proficiency may struggle to use applications without proper inclusive and accessible designs [12,17]. There is a paucity of research of socioculturally tailored digital health interventions that incorporate design based on input from minoritized populations [13]. Example results of engagement in design include actors who are relatable to the user population in photographs and videos [18]. Communication patterns and health literacy levels are important considerations, as persons with low health literacy have higher rates of chronic disease than those with adequate literacy [19]. Prior user-centered design work crafted motivational text messages in English and Spanish for persons with low literacy levels to increase physical activity [20]. A scoping review of digital health studies found that persons with disabilities were rarely included, leading to non-accessible applications [21].

A limited, but increasing, number of studies investigate technology implementations in disadvantaged populations. Effective interventions must encompass acceptability (through user acceptance testing), practicality, and be designed to address the needs of at-risk populations [22,23]. The emergence of equity-focused digital health research models have helped improve the delivery of more inclusive healthcare. The adoption-focused Integrative Model of eHealth Use was updated to include health literacy and computer literacy, and the eHealth Equity Framework incorporates technology access, material circumstances, and societal values [24,25]. Health equity factors such as poor engagement with digital health, health policies, and digital determinants of health (e.g. digital literacy, lack of internet access) are examined in the Digital Health Equity Framework, which focuses on access and adoption [26]. An equity-focused behavioral science model called the ConNECT Framework integrates context, fosters inclusion, and prioritizes training of healthcare providers and implementors to improve health equity [27]. The above models include incomplete equity concepts in relation to implementation, focusing on a particular phase or aspect of an implementation. This paper therefore develops a broader implementation model that increases focus on causes of inequities in digital health implementations from planning to outcome tracking.

Few equity-focused implementation models exist to date though there are many general implementation frameworks available to researchers [28,29]. The Equity-Focused Implementation Research for Health Programs (EquIR) model was developed in 2019 and enables researchers and executives to incorporate equity considerations during the entire implementation process from the initial planning phase through to evaluation [30]. EquIR’s from-planning-to-outcome scope, as well as its focus on providing solutions to equity challenges sets it apart from the Consolidated Framework for Implementation Research (CFIR) [31]. The CFIR model is a determinants framework; meaning it was designed to guide the systematic evaluation of factors influencing implementations of programs in various settings in the domains of intervention characteristics, outer setting, inner setting, characteristics of individuals, and process. The CFIR model was updated to include human-equality centeredness, but does not include steps informing how to alleviate said inequalities [30,31]. Besides EquIR and CFIR, other equity-focused implementation frameworks include The Transcreation Framework and the Health Equity Implementation Framework [22,28].

EquIR was designed by implementation researchers, health equity experts, and results from a systematic review. It is cyclical in nature; beginning with equity-focused population health status and ending with a new post-implementation population health status. It includes five progressive phases: population assessment, planning, designing, implementing, and equity-focused implementation outcomes [30]. Each phase includes direction for incorporating equity issues and approaches to diminish them. As it is a relatively new model, the applications to date are limited. However, the EquIR framework was used to investigate challenges that impact gerontology researcher-community partnerships and to generate potential solutions to obstacles [32].

There is a need for a digital health implementation model that similarly supports equity-focus throughout all phases of digital health planning and implementation. The purpose of this paper is to modify EquIR to include constructs from digital health implementation frameworks, thereby creating recommendations on the equitable implementation of new digital health technologies.

## Methods

The digital health and equity-focused implementation model (DH-EquIR) builds on the original EquIR model by integrating digital health implementation into the conceptualization of equitable implementations. DH-EquIR was designed by identifying patient and program-related factors contributing to the successful implementation of digital health technologies. We conducted a narrative review following the Scale for the Assessment of Narrative Review Articles [33] to comprehensively describe and interpret previously published literature on digital health implementation models incorporating concepts of health equity [34]. A literature synthesis table was created with each EquIR construct as a header. As each digital health implementation study was reviewed, digital health concepts that related to constructs from the original EquIR model were inserted into the literature synthesis table. These patient- and program-related implementation factors were then fused into the EquIR model to create an adaptation capable of guiding equity-focused digital health implementations. The lead author conducted the primary synthesis of results. The other authors, who all have extensive knowledge of the material, then gave feedback on the contents.

### Search strategy

Articles were found via database searches, reference lists, expert literature, and current practice guideline review. Literature searches (PubMed, CINAHL, Google Scholar) were conducted August 2022 through May 2023 and included medical subject heading (MeSH) terms: *Cultural Diversity*, *Minority Groups*, *Minority Health*, *Healthcare Disparities*, *Health Status and Disparities*. Additional search terms included: *health equity*, disparit*, *health technology*, *digital health*, *mhealth*, *adoption*, *conceptual framework*, *conceptual model*, *implementation science*, *implementation research*, and *translational research*. A medical librarian was consulted for the search strategy. The MEDLINE/PubMed Health Disparities and Minority Health Search Strategy [35] query was used in combination with search terms: *Mobile Applications [Mesh]*, *Telemedicine [Mesh]*, *Remote Sensing Technology [Mesh]*, *“digital health"[TW]*, *"mhealth"[TW]*, *"virtual health"[TW]*, "smartphone*"[TW], and “implementation*”[TW].

The “D&I Models in Health Research and Practice” website (https://dissemination-implementation.org) was used to explore theories, models, and frameworks that involved both health equity and implementation research.

### Inclusion and exclusion criteria

The search included English language articles published from January 2017 through May 2023; international studies were included. Primary research, systematic reviews, and scoping reviews covering health equity, implementation frameworks, and digital health implementations were included. Individual primary research studies were required to involve patients as the focus. Exclusion criteria omitted clinician-focused articles, conference abstracts, editorial and opinion articles, magazine articles, and protocol proposals.

### Conceptual model

Equity-focused recommendations and observations were extracted from the available articles and organized by the original EquIR model’s constructs, which may be viewed in its original publication [30]. Digital health planning-related factors were those related to the identification of disadvantaged groups, the quantification of health inequities, and the identification of equity-sensitive recommendations. Similarly, relevant digital health literature was incorporated into the process of designing an intervention and the identification of key actors, barriers, and facilitators to equity-focused recommendations. The implementing EquIR phase was augmented with concepts from digital health implementations including communication plans, the definition of resources and incentives, strategies to overcome barriers, and monitoring and evaluation strategies for digital health. Equity-focused implementation outcomes included digital health monitoring metrics and strategies. While keeping the original EquIR model’s visual structure, the DH-EquIR model revised the constructs to be digital health equity implementation-focused based on the data extracted from the source articles.

## Results

### Adapted conceptual model

**Fig 1:**
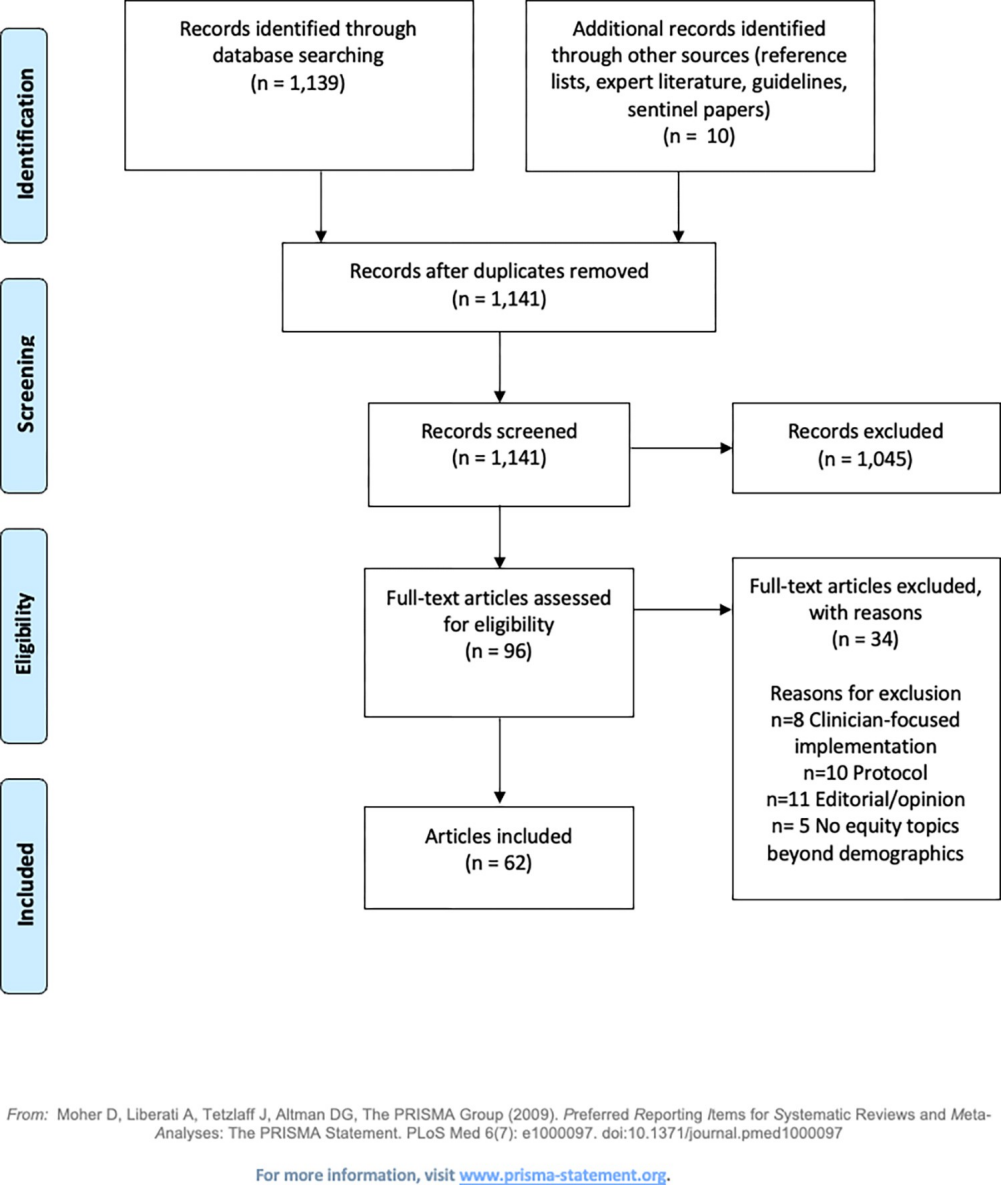
–PRISMA Diagram.

The Preferred Reporting Items for Systematic Reviews and Meta-Analyses (PRISMA) diagram is shown in Fig 1. Sixty-two articles were reviewed including existing digital health equity conceptual models and frameworks, digital health implementation models and frameworks, research articles, guidelines, and concept analyses. Digital health models included CFIR, the Technology Acceptance Model (TAM), unified theory of acceptance and use of technology (UTAUT), and the Integrated Technology Implementation Model (ITIM) [36–38]. Health equity models included the Digital Health Equity Framework (DHEF), the Framework for Digital Health Equity, and the Equity Checklist for Health Technology Assessment (ECHTA) [26,39,40]. Research articles include systematic reviews of barriers and facilitators to digital health uptake, interventions to increase technology usage by vulnerable populations, and articles on inclusive design. Table 1 includes details around implementation models and equity tools that were used to construct the DH-EquIR model. Table 2 depicts concepts taken from each source as they relate to the original EquIR model, as well as implications for future research identified in the articles. Table 3 includes exemplars of studies investigating equity issues within digital health implementation studies. The 17 exemplars were selected based on their inclusion of diverse patients and equity-focused recommendations. Additional specific examples of digital health implementation studies may be referenced in a scoping review of digital health interventions in ethnic-minoritized populations [41], a systematic review of interventions to increase patient portal use [42], and a systematic review of health and wellness technology use by historically underserved health consumers [43].

**Table 1:** Analysis of models, reviews, and equity tools: Relevant aspects included in the development of the DH-EquIR model.

Lead Author, Year	Type / Model / Tool	Aspects of equity	Key Concepts for DH-EquIR Development
Benkhalti 2020	Checklist to guide equity considerations	Checklist to include equity considerations in health technology assessment	• Define “equity of what?” (need, access, health status, function, prognosis, quality of life, SDOH) • Define subgroups of target population • Consider whether technology could lead to biases for or against specific populations • Engage disadvantaged community members as leaders to help define inequities
Ramasawmy 2022	Scoping review	Inequality constructs map onto individual, provider/health care system, population/society, and intervention levels of action	• Digital literacy, internet access, hardware • Lack of trust, fear of discrimination • Experiences with culturally insensitive clinician behavior, communication barriers, racism
Richardson 2022	Framework for digital health equity	National Institute on Minority Health and Health Disparities Research Framework Expanded for Digital Health Equity	• Individual, interpersonal, community, and societal levels of influence of digital environment
Latulippe 2017	Literature review	Strategies to reduce social health inequalities	• Respect cultural attributes of future users • Strategies to overcome barriers (subsidize phone plans, Wi-Fi hotspots, renting programs) • Translation features • Accessible technologies • Use of video instead of written materials for persons with low literacy skills • Volunteers to help older people with digital health
Borsci 2018	Human and Economic Resilience Design for Medical Technology model	Evaluation of unmet needs for technology development, human factors/user experience, realistic scenario development, monitoring/evaluation strategies	• Value engineering • Economic evaluation
Gemert-Pijnen 2011	Center for eHealth Research model	Socioeconomic	• Human-centered design principles • Value specification
Espay 2019	Roadmap for the development of digital health tech	Not applicable	• Determine benefit-to-burden ratio, digital health should be unobtrusive to patients • Solution should be visually intuitive • Interoperability • Need for sustainable financial model • Perceived privacy
Schoville 2015	Integrated Technology Implementation Model	Not applicable	• Facilitator (champion) that guides implementation • Leadership–executives, managers, and consultants that promote technology adoption
Hess 2010	Equity Implementation Model	Model suggesting evaluation of individual and comparative net benefits of technology influencing intention to use	• Equity-self, equity-authority figure (does the clinic/org gain more than the patient), equity-other users (how patient compares self to others) • Acceptability • Adoption
Davis 1989	Technology Acceptance Model	Not applicable	• Perceived usefulness/define monitoring and evaluation strategies • User experience scales/acceptability
Venkatesh 2013	Unified Theory of Acceptance and Use of Technology	Not applicable	• Behavioral intention to use technology/acceptability
Damschroder 2009	Consolidated Framework For Implementation Research	Not applicable	• Implementation research article on which original EquIR model is focused • Structure for identifying influences on implementation • Approaching complex, multi-level, interacting constructs key to implementation
Bodie 2008	The Updated Integrative Model of eHealth Use	Health literacy	• Measurement of literacy • Health, digital literacy • Trust
Antonio 2019	eHealth Equity Framework	Unmet needs analysis, planning action on social determinants of health	• Identify inequities as socially-mediated factors • Considering how digital health can address health inequities
Alcaraz 2017	ConNECT Framework	Integrating context, inclusion, equitable diffusion of innovations, communication, and specialized training	• Population health status: intersectionality and health disparities • Importance of culturally, contextually, and linguistically relevant content • Specialized training to ensure broader transcultural relevance
Nápoles 2018	The Transcreation Framework	Planning and delivering interventions to reduce health disparities by resonating with targeted community	• Integrating with community’s knowledge, local programs, and participants from the beginning • Hiring and training community members as interventionists and study staff • Methods for assessing implementation processes • Sustaining effective programs by building community capacity
Woodward 2019	Health Equity Implementation Framework	Societal influence (economies, policies, sociopolitical forces)	• Societal influence on implementation (economies, policies, sociopolitical forces) • Assessing implementation barriers on the innovation, organizational, and patient levels • Collecting data on facilitators: application-based reminders, building trust, eagerness for education

**Table 2:** Digital Health Implementation Source Article Concepts Mapped to EquIR Concepts and Implications for Future Research.

EquIR-related concepts from source articles	Extracted Components
Population health status [24,26,27,39]	• Social determinants of health • Digital determinants of health
Research question [22,24,40]	• Define: equity of what? • Define disadvantaged groups • Could technology lead to biases for or against specific population groups?
Identify disadvantaged groups [24,41,44–46,67]	• Financial barriers/low income/lack of housing • Older adults • Rural • Ethnic minoritized populations • Unequal access to hardware, software, internet • Digital literacy • Low level of education • Persons with disabilities • Sexual orientation
Quantify current health inequities [27,40,47,49,56]	• Insurance coverage • Health literacy assessments • Area Deprivation Index • Rural Urban Commuting Area (RUCA) codes to classify rurality • American Community Survey (ACS) to estimate proportion of adults who attained at least a high school degree • Define what constitutes inequities in the context of the region through interviews with engaged community members
Develop or identify equity sensitive recommendations [27,42,44,45,47,51–55,80]	• Software ease of use • Training and assistance programs • Identify stressors and incorporate into scenarios for technology development • Define device features that ensure resilience • Appreciate differences between specialties • Tailor to the target regions • Human-centered design
Identify key actors for implementing equity-focus recommendations [22,38,40,51,59]	• Clinicians, payers, managers, patients, caregivers • Engaged community members from disadvantaged populations • Leadership–executives, consultants • Regulatory approval • Government subsidies/grants, insurance coverage • Facilitator/champions • Vendors/engineers • Community health workers • Community advisory boards • Patient advisory committees
Identify barriers & facilitators to equity focused recommendations [22,24,25,28,41,44,45,51,52,60–63]	• Digital literacy, internet access, hardware • Loaner devices • Credibility • Incorrect beliefs about medical condition • Preference for in-person care • Increased time and effort • Fear of using digital health • Lack of validation of accuracy • Trust, fear, discrimination, culturally insensitive behavior • Tools that respect cultural attributes of users
Design a communication strategy with equity focus [25,27,46,55,67]	• Advertisements • Social reference • Patient newsletters, patient-centered social media groups, community centers, and disability groups
Define resources and incentives [44,60–62]	• Health benefits • Reassurance, decrease anxiety • Self-manage condition • Minimize overtreatment and maximize quality of life • Reduce travel
Design strategies to overcome the identified barriers [22,27,28,42,43,45–47,51–53,57,59,68–71,73]	• Technology support • Balance burden and accuracy • Visually intuitive • Test low-fidelity prototypes • Subsidize technology and internet • Asynchronous technology (should not require continuous internet access) • Translation features, culturally sensitive content • Transcreation process to culturally tailor interventions • Accessibility for people with disabilities (WCAG AAA guidelines) • Use cellular devices as opposed to Bluetooth for built in connectivity • Use video instead of written materials • Simplify text, graphics, audio • Volunteers to help older people learn digital health
Define the monitoring and evaluation strategies [22,36,42,51–53]	• Clinical pathway mapping • User experience scales • Unmet needs analysis • Estimate cost effectiveness • Evaluate for data errors as result of user error • Web and mobile analytics software • Acknowledge that the impact of digital health is indirect compared to pharmaceuticals
Sustainability [38,51,59,62,75,80]	• Potential of text-message fatigue, individual turning off alerts • Interoperable platform • Sustainable financial model
Coverage [81]	• Telemedicine • RPM • RTM • Medicaid- uneven RPM coverage • Problems with copays
Implementation Cost [62]	• Cost savings and efficiency from using the program
Fidelity [45,51,56,62]	• Easy reminders • Personalized components • Improved communication with healthcare providers
Feasibility [28,42,43,45,51,56,61,67,70]	• Access to cellphone and devices • Complexity of the digital health application • Financial means to purchase devices and service • Friends or family available for help
Appropriateness [61,62,67,75,76]	• Attitude towards digital health • Digital literacy • Illness perception and social context (patient feels physical functioning does not align with goals of intervention) • Older adults’ perception of messages as condescending • Older adults’ mistrust over quality of care via digital health • Poor dexterity and anxiety using technology
Adoption [61,62,67,75,76,80]	• Service delivery time, service quality, result demonstrability • Community-based support and a social learning environment • Personalized, comprehensive education • Text messaging to establish a routine • Two-way messaging with providers • Easy to integrate into daily routine • Relationship with healthcare providers
Acceptability [36,44,51,57,59,62,67,68,75,77,82]	• Individual and comparative net benefits of technology influencing intention to use • Perceived usefulness, perceived ease of use, behavioral intention to use, actual system use • Compatibility, self-efficacy, training, habit • Performance expectancy, effort expectancy, social influence • Perceived privacy • Perceived cost

**Table 3:** Equity issues in exemplar digital health implementation studies.

Lead Author, Year	Equity consideration	Disadvantaged population
Alwashmi 2020	Software ease of use, training/assistance, credibility of digital health	Low digital literacy, older age, financial barriers, lack of internet
Castillo 2022	Culturally tailored content, translations	Spanish-speaking Latino immigrants living in the US
Hincapie 2019	Cost, insurance coverage, personalization, engagement, software ease of use	Low-income, limited access to technology and internet
Hossain 2019	Advertisements, social reference, attitudes toward digital health	Rural (Bangladesh), age, gender, education
Kirkland 2020	Training and assistance programs, device loan programs, provide internet connectivity via cellular devices	Race, low-income, rural
Javier 2019	Training and assistance programs, health literacy assessments, quantify health inequities (RUCA and ACS)	Race, ethnicity, rurality, education level
Johnson 2020	Barriers: incorrect belief about medical condition, increased time and effort, physical dexterity, distrust Incentives: minimize overtreatment, maximize quality of life	Older age (the sample was highly educated with 79% with a bachelor’s degree and 97% white)
Li 2022	“High tech, high touch” model, community health worker involvement, adoption	Ethnicity, rurality
Mantini 2020	Feasibility, strategies to overcome barriers, provision of devices	Race, ethnicity, rurality
Park 2020	Sustainability: text message fatigue, disinterest, turning off alerts	Older age (the sample was mostly white (74%), college graduate (75%) and male (75%))
Pekmezaris 2020	Maximizing acceptance with cultural tailoring, improving translations	Ethnicity, language for care
Russ 2020	Recruiting a diverse sample for research, communication strategies, translations, culturally-sensitive content	People with disabilities, race, ethnicity
Russell 2021	Identifying barriers (distance, time)	Transgender and gender diverse minors and young adults
Ruvalcaba 2019	Culturally tailored interventions, transcreation process, strategies to overcome barriers	Spanish-speaking Latino patients
Slevin 2019	Digital literacy, community-based support, education, appropriateness	Technology ownership, education level, occupational status
Valdez 2021	Strategies to overcome barriers, recommendations for improving accessibility of digital health	People with disabilities
Wang 2020	Identifying barriers, strategies to overcome barriers, educational programs/communication	Insurance, low-income

**Fig 2:**
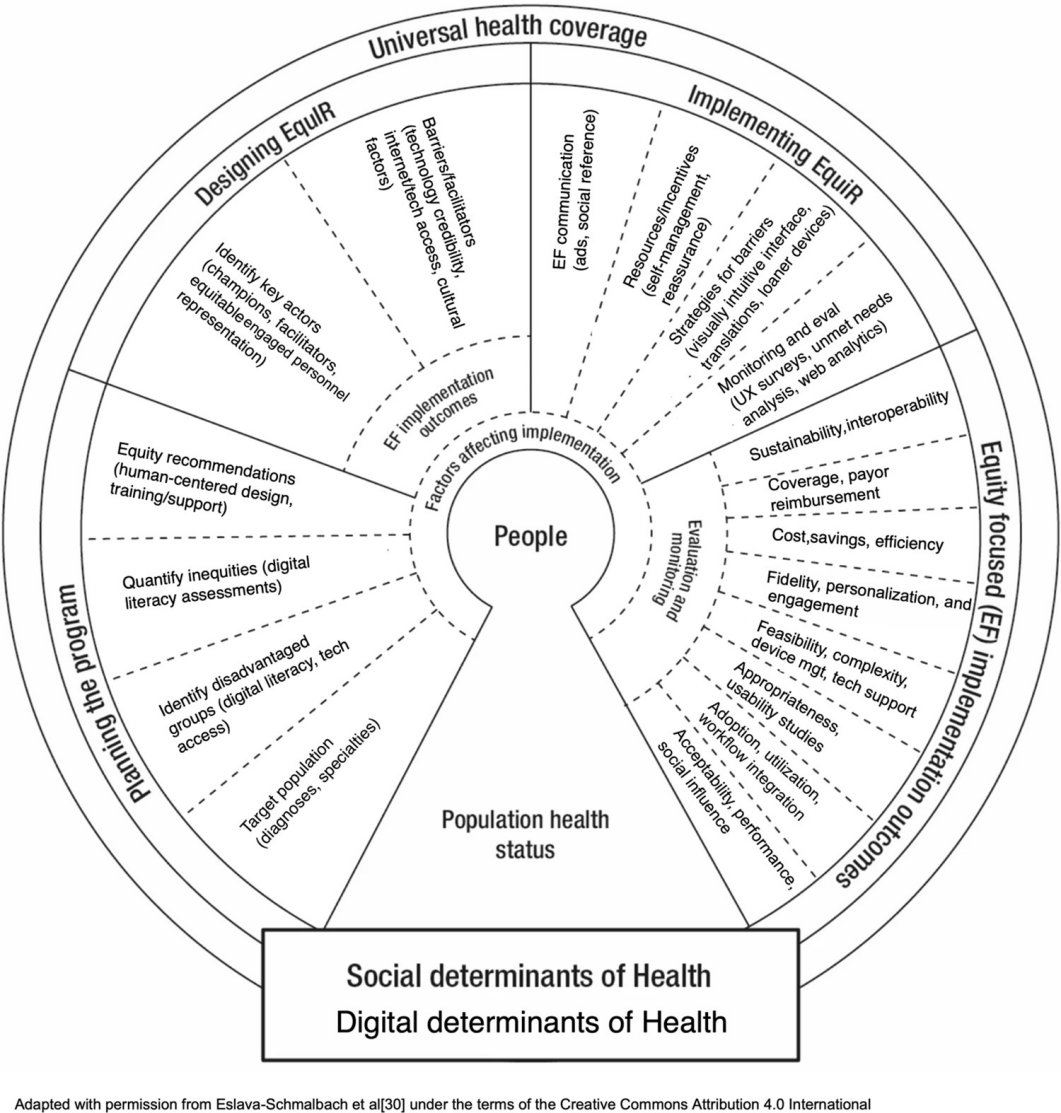
Digital Health Equity-focused Implementation Research Model (DH-EquIR).

The DH-EquIR model (Fig 2) was created by incorporating the digital health concepts from the literature review into the corresponding location on the original EquIR model figure. In line with the original EquIR model, the DH-EquIR model contains five phases: assessing population health status and SDOH/DDOH as a starting point, planning the program, designing the program, implementing the program, and equity-focused implementation outcomes. The model culminates with a reassessment of the population health status post implementation. The text below describes each phase of DH-EquIR to give additional context.

### Population health status and determinants of health

Population health status of both the general population and the disadvantaged population should be taken into account. Population health status guides the researcher in broadly considering the inequities in a population before an intervention is designed and implemented, and evaluating how those inequities are improved as a result of a program [30]. Relatedly, SDOH such as occupation, gender, poverty, race/ethnicity, and socioeconomic condition are typically included in an assessment of population health status [30]. Digital determinants of health (DDOH) refer to access to technology, digital literacy, and community infrastructure (e.g. broadband internet) [26,39]. The DH-EquIR model emphasizes the combined importance of SDOH and DDOH in assessing population health.

### Planning the program

#### Target population

Identification of relevant research questions and the target population of interest should be the first step of planning.

#### Identify disadvantaged groups

Within the target population, identify which disadvantaged populations will potentially be impacted by the digital health implementation. Disadvantaged groups for digital health frequently include financially disadvantaged, older adults, rural populations, racial and/or ethnic minoritized populations, persons with low digital literacy, persons without access to devices and the internet, low levels of education, and persons with disabilities [41,44–46].

#### Quantify current health inequities

The population health status starting phase is the general population; while in this step work is done to more specifically measure health inequities in the target population. Methods of quantifying health inequities from the literature include digital literacy assessments, health literacy and limited English proficiency assessments, estimates of proportion of adults with at least a high school degree, and interviews with engaged community members [40,47–49]. Implementations require consideration of subgroups that will use the technology, as well as an assessment of potential biases for or against specific population groups (e.g. identifying under-represented groups not included as engaged partners that should be) [40]. The appraisal may include collecting demographics and a SDOH screener. Inequities may be identified by using a framework such as PROGRESS Plus (place of residence, race/ethnicity/culture/language, occupation, gender/sex, religion, education, socioeconomic status, and social capital) [50]. Additional DDOH can be assessed by surveying specific populations about smartphone ownership and internet access, or by leveraging community-level data to examine structural barriers such as broadband internet availability. Collecting this data helps determine potential inequities in an implementation so that strategies may be developed. Note that many other assessment and measurement tools are available, this article mentions those tools found during our literature review.

#### Equity-sensitive recommendations

Program recommendations for equity-sensitive digital health implementations include focus on software ease of use, training and assistance programs, realistic scenario development, human-centered design principles, and tailoring technologies to target regions [42,44,45,47,51–54]. In some cases, it may be appropriate to collaborate with social benefit programs to reach patients [55]. Cost and payor coverage must also be considered during planning [56]. Implementers should also consider community and government resources to support digital health projects such as the United States Health Resources and Services Administration’s Telehealth Resource Centers. Digital health design activities should prioritize the inclusion of disadvantaged populations that may benefit from a solution, as opposed to the mid-to-high socioeconomic users who are frequently recruited to provide feedback on initial designs. Models such as ADAPT-ITT tailor existing applications to meet the needs of target populations [57,58]. The equity checklist for health technology assessment includes additional considerations when developing a new solution [40].

### Designing the program

#### Identify key actors

The identification of key actors for digital health implementation include engaged personnel who will support the implementation, regulatory approval contacts for some forms of digital health (e.g. telemedicine visits), evaluating and contacting payors for coverage information, considering contacts for government subsidies for those who need devices or access, and securing buy-in from healthcare executives [38,40,51,59]. Other roles that facilitate a successful implementation include effective champions and facilitators to guide the project, and responsive design and engineering teams to develop the solution [38]. It is recommended to include members from under-represented groups as engaged partners in development. Note that key actors may potentially contribute to either barriers or facilitators, depending on their view of digital health.

#### Identify barriers and facilitators to equity-focused recommendations

Potential barriers include fear of using digital health, lack of validation of accuracy of digital health, preference for in-person care, lack of care team resources, increased time and effort, and previous experiences of culturally insensitive behaviors and communication barriers [40,41,51,60–62]. Digital health may eliminate transportation requirements and increase time savings due to its remote nature and convenience [63]. Patients reported they would be more likely to use digital health if it was recommended by their provider; a facilitator of digital health credibility [44]. Including community health workers in digital health programs has shown to enable “high tech, high touch” implementation approaches [64], while community advisory boards and patient advisory committees reduce intimidation and facilitate community input [57]. In the US, India, and other countries, community health workers are a supportive role filled by persons who are not typically health professionals; they serve as a frontline advocates and educators, bridging the gap between healthcare services and underserved populations to promote and improve overall community health [65, 66]. Digital health’s ability to improve communication between patient and clinician, technology support from family or friends, and potential for saving time are other major facilitators [61]. Another facilitator is the creation of digital health tools that respect the cultural attributes of patients; such as making the tool available in languages other than English [45].

### Implementing the program

#### Design an equity-focused communication strategy

Digital health programs were found to be most effectively communicated with social reference and advertisements [67]. Social reference describes the referral of a product or service by someone an individual trusts. Advertisements may help to establish a digital health tool’s credibility. Placing adverts in patient newsletters, patient-centered social media groups, community groups, and disability groups are other communication strategies [46,55].

#### Define resources and incentives

Resources and incentives for digital health include possible health benefits for patients, reassurance from a connected care team, increased ability to self-manage a condition, and reduced travel [44,60,62]. The incentives will differ based on the target population (e.g diagnoses, medical specialties) and intervention. One example of an incentive is better control of hemoglobin a1c and long-term comorbidities in patients with diabetes.

#### Define strategies to overcome the identified barriers

Implementation teams should design strategies to overcome the barriers identified in the previous phase. Strategies include the development of visually intuitive solutions, provision of technology support, subsidization of smartphone plans and technology renting programs, inclusion of translation features, and development of accessible software interfaces [42,45–47,51,59,68–70]. Other strategies include using video instead of written materials to increase comprehension among persons with low literacy skills, simplifying text, and creating tools that respect the cultural attributes of users [43,45,47]. The transcreation process may be used to culturally tailor interventions [71]. New methodologies for precision cultural tailoring have been introduced, while others promote a better goal as usability and accessibility across multiple cultural groups [72]. To improve accessibility of applications, compliance with technical accessibility guidelines (such as appropriate font size and color contrast) is encouraged [57,73]. Issues around device and internet access may be addressed with device loaner programs and the use of cellular devices which do not require individual data plans [52].

#### Define monitoring and evaluation strategies

Engaging targeted end-users in developing outcomes relevant to them is an important best practice to utilize when defining evaluation and monitoring strategies [74].The impact of digital health is indirect compared to interventions such as pharmaceuticals [53]. Clinical pathway mapping defines the clinical contexts in which new technology will be used; this may serve as a foundation for health economic evaluation [53]. Other outcomes include user experience and patient satisfaction surveys, the estimation of cost effectiveness, and completion of an unmet needs analysis to support redesign of the intervention [36,53]. Digital health should be routinely evaluated for data errors as a result of human error [51]. Finally, web and mobile analytics software can be used to track engagement [42].

### Equity-focused implementation outcomes

#### Sustainability and interoperability

Digital health sustainability is enabled with sustainable financial models and interoperable platforms [38,51,59,62]. Interoperable platforms improve sustainability by removing workflow inefficiencies that drive abandonment of digital health initiatives and costly integrations that prevent many practices from sustaining digital health tools. Interoperability also impacts other implementation outcomes.

#### Coverage and payor reimbursement

Coverage involves the degree of reach, access, or effective coverage on the disadvantaged population [30]. For example, the proportion of eligible target population members who participated in the digital health program. Separately, reimbursement coverage could be impacted by Medicare, Medicaid, and private insurance coverage for particular technologies in the US. Even if not directly reimbursed by payors, implementation cost can be offset by cost savings and efficiency from digital health [62].

#### Implementation costs, cost savings and efficiency

Implementation costs should be tracked, while outcomes such as improved efficiency, avoided emergency room visits, and decreased hospital readmissions can indicate cost savings. It is also important to monitor for associated costs such as additional workforce needed to maintain the program [62].

#### Fidelity, personalization, and engagement

Fidelity is improved with patient engagement features such as easy reminders, personalized components, and improved communication with healthcare providers [42,51,56,62]. Key performance indicators may include utilization rates of features such as personalized education and clinician messaging.

#### Feasibility, device management, and technical support

Feasibility is impacted by complexity of the application, the support of friends and family, and financial factors [42,43,56,61]. The complexity of the application can be measured with usability reviews.

#### Appropriateness, satisfaction surveys, and usability studies

Appropriateness can be measured with digital literacy assessments, satisfaction surveys, and ease of use surveys [62,67,75].

#### Adoption, utilization, and workflow integration

Adoption is improved with personalized education, ease of integration into daily routines, and two-way messaging with care teams [61,62,5,76]. Acceptability includes perceived usefulness, behavioral intention to use, compatibility, perceived privacy, and perceived cost [36,37,67,77]. Integration of self-monitoring data into the care team’s existing workflows enables personalized feedback to be given to the patients [64]. The act of monitoring and responding to clinical data requires a dedicated team member at the clinic, or provisions to incorporate the extra required work into a provider’s limited time.

#### Acceptability, performance, and social influence

Acceptability or effectiveness is another important outcome. Web and mobile analytics software as well as qualitative inquiry can help to measure performance, social influence, and usefulness of a digital health intervention [42].

## Discussion

To the authors’ knowledge, the DH-EquIR framework is the first to integrate digital health implementation concepts into the EquIR model. This is significant because conceptual models and frameworks to date are very limited in the digital health equity domain. The DH-EquIR framework serves to pragmatically guide the planning, design, implementation, and monitoring of implementation studies that deploy digital health tools into practice within underserved populations. The inclusion of digital determinants of health is an example of an adjustment to the original model. While it is still critical to evaluate SDOH throughout the implementation process, DH-EquIR encourages researchers to further consider elements such as digital literacy and access to internet. Additional elements for DDOH may include accessible content needed by persons with disabilities, or assessments of smartphone addiction in which strategies to maintain reduced screen time may be required. The inclusion of human-centered design principles in the development of equity-sensitive recommendations promotes co-design with members of underserved populations. In the identify key actors stage, the DH-EquIR model highlights the importance of including underserved population members as organizational leaders to balance power dynamics [40]. Additionally, user experience surveys and patient engagement concepts broaden the scope beyond EquIR’s original bounds. The DH-EquIR framework serves to drive implementation planning and execution questions. Its practical nature allows the researcher to reference the model for ideating around potential barriers as well as solutions. The model is intentionally broad in nature, which presents challenges around specificity. Digital health applications vary in scope and complexity; the model may need to be adjusted for specific use cases.

Given the proliferation of digital health applications and the increasing reliance on internet access in daily life, it is critical to address health equity. Due to SDOH and DDOH, there are many barriers to universal digital health coverage. Without intentional consideration of health equity, digital health implementations skew towards usage in younger, wealthier populations that can afford to pay a premium on the latest devices and applications. Women, minority ethnic groups, and older people are frequently underrepresented in research, resulting in non-generalizable findings across populations [78]. Underrepresentation of these groups in digital health research studies also means that they were not included in the design of the application or device. The introduction of generative artificial intelligence into digital health increases the need for equity vigilance, as AI can perpetuate bias at scale [79]. Digital health developers and implementation specialists can use DH-EquIR to avoid repeating the same mistakes that have previously led to inequities. Planning for digital health equity improves the overall quality, usability, and allows the needs of diverse communities to be met.

Further, digital health tool development is a participatory process and involves continuous evaluation cycles intertwined with implementations that change the organization of healthcare [54]. The DH-EquIR model incorporates these tenets, as it visually depicts the cyclical nature of the development and implementation lifecycles. The DH-EquIR model can be used to identify key dimensions and factors to consider in their research study, as well as serve to remind researchers to include health-equity planning in every phase of implementation.

### Limitations

The DH-EquIR model has several limitations. First, the model requires empirical testing before being used in operational contexts. The added concepts require real world testing in deliberate studies designed to test the model’s feasibility. Only patient-focused (technologies designed for patients as opposed to clinicians) studies were included in this analysis. In addition, a single author (LG) completed the literature search, article screening, and determination of article inclusion. Due to resource restraints a higher-level review such as systematic review could not be undertaken. Other gaps include the need for further methods of measuring inequities in digital health and enforcement of standards to enable interoperable programs.

## Conclusion

Historically, digital health has not been inclusive of the needs of diverse populations. The digital divide continues to disproportionally benefit individuals not impacted by barriers such as low income, poor internet access, and lack of necessary devices. Health equity research has produced a number of helpful models and frameworks to address inequities, yet a comprehensive model incorporating digital health implementation needs from planning to measuring outcomes was lacking. The DH-EquIR model synthesized concepts from health equity models and combined them with concepts from equity-focused digital health implementation research. The model drives equitable technology implementations beginning with assessing the target population, and progressing through planning the program, designing the program, implementing the program, and measuring equity-focused implementation outcomes.
